# Aggregation of Human Mesenchymal Stromal Cells Eliminates Their Ability to Suppress Human T Cells

**DOI:** 10.3389/fimmu.2020.00143

**Published:** 2020-02-25

**Authors:** Anthony J. Burand, Lin Di, Lauren K. Boland, Devlin T. Boyt, Michael V. Schrodt, Donna A. Santillan, James A. Ankrum

**Affiliations:** ^1^Roy J. Carver Department of Biomedical Engineering, College of Engineering, University of Iowa, Iowa City, IA, United States; ^2^Fraternal Order of Eagles Diabetes Research Center, University of Iowa, Iowa City, IA, United States; ^3^Department of Obstetrics and Gynecology, Carver College of Medicine, University of Iowa, Iowa City, IA, United States; ^4^Center for Immunology and Immune Based Diseases, Roy J. and Lucille A. Carver College of Medicine, University of Iowa, Iowa City, IA, United States; ^5^Center for Hypertension Research, University of Iowa, Iowa City, IA, United States

**Keywords:** mesenchymal stem cells (MSCs), budesonide, PGE2, synergy, T cell, proliferation, immunosuppression

## Abstract

Mesenchymal stromal cells (MSCs) are administered locally to treat sites of inflammation. Local delivery is known to cause MSCs to aggregate into “spheroids,” which alters gene expression and phenotype. While adherent MSCs are highly efficient in their inhibition of T cells, whether or not this property is altered upon MSC aggregation has not been thoroughly determined. In this study, we discovered that aggregation of MSCs into spheroids causes them to lose their T cell-suppressive abilities. Interestingly, adding budesonide, a topical glucocorticoid steroid, alongside spheroids partially restored MSC suppression of T cell proliferation. Through a series of inhibition and add-back studies, we determined budesonide acts synergistically with spheroid MSC-produced PGE2 to suppress T cell proliferation through the PGE2 receptors EP2 and EP4. These findings highlight critical differences between adherent and spheroid MSC interactions with human immune cells that have significant translational consequences. In addition, we uncovered a mechanism through which spheroid MSC suppression of T cells can be partly restored. By understanding the phenotypic changes that occur upon MSC aggregation and the impact of MSC drug interactions, improved immunosuppressive MSC therapies for localized delivery can be designed.

## Introduction

Mesenchymal stromal cells (MSCs) have been used for decades to treat inflammatory diseases because of their ability to produce a range of immunosuppressive products. MSC produced IDO (1), kynurenine (2), PGE2 (3, 4), CD73 (5, 6), TGF-β (7), IL-6 (8), and TSG-6 (9), among others, enable MSCs to modulate the behavior of T cells, B cells, macrophages, natural killer cells, dendritic cells, and neutrophils (10). The ability of MSCs to both dampen inflammation and promote an anti-inflammatory tolerogenic phenotype in multiple immune subsets has made them candidates to treat complex inflammatory diseases, such as graft vs. host disease, Crohn's disease, multiple sclerosis, asthma, chronic wounds, and rheumatoid arthritis.

The vast majority of techniques to evaluate and screen MSC potency rely on *in vitro* adherent cultures of MSCs; however, this may not reflect their environment post-transplantation. Currently, about half of all clinical trials utilizing MSCs have locally injected MSCs (11). Local delivery of MSCs places MSCs near the site of injury or inflammation while eliminating the risk of embolization that comes with systemic infusions (12, 13). However, when MSCs are injected into a spatially confined site they are known to aggregate to form spheroids. This aggregation phenomenon has been observed in rodents after intraperitoneal (14), subcutaneous (15, 16), and intraventricular (17) injections, and alterations in MSC phenotype have been observed in intramuscular injections (15), prompting the study of the spheroid MSC phenotype. While it is known that MSCs in spheroids dramatically shift their gene expression upon local injection (14), the full consequences of aggregation on MSC interactions with T-cells is not known. The frequent utilization of local injection and evidence of transcriptional changes upon MSC aggregation challenges the use of adherent MSC potency assays to evaluate MSC products before use in local applications.

Alterations in secretome change MSCs interactions with immune cells. Studies to date have shown aggregation into spheroids causes MSCs to upregulate PGE2, TSG-6, IL-1α/β, and STC1, as well as several matrix factors (14, 18, 19). In trans-well experiments with immortalized mouse macrophages, human spheroid MSCs reduced macrophage production of TNF-α and increased IL-10 more than adherent MSCs (14, 18, 20). Similar enhanced interactions of spheroid MSCs with macrophage populations have been observed with THP-1-derived macrophages as well as a mouse model of peritonitis (14, 18). In animal models, spheroid MSCs have been shown to reduce spontaneous limb loss in a hind limb ischemia model (21), to lower infarct volume in a stroke model (22), to rescue kidney cells from apoptosis (23), and to enhance bone regeneration (24), possibly due to enhanced levels of growth factor secretion (25). While appearing beneficial in several settings, the full impact of aggregation on MSCs' immunomodulatory phenotype has not been revealed, which is critical both for suppression of inflammation and, in allogeneic uses, immune evasion (26).

Many of the disease indications in which localized injection of MSCs occur are mediated by T cell recruitment and effector function. This is because adherent MSCs strongly suppress activated T cells, PBMCs, and mixed lymphocyte reactions (MLR) *in vitro* (1, 2, 27–30). However, few studies to date have looked at the interactions of non-differentiated spheroid MSCs with T cells. To translate MSCs to the clinic, we must know if spheroid MSCs have comparable immunomodulatory potency to their adherent counterparts, or if they display an entirely different, not necessarily superior, immunomodulatory profile. If these profiles are distinct, it makes little sense to study MSC potency under adherent conditions or use adherent potency assays to screen MSC products, since their behavior shifts rapidly upon local injection.

Herein, we aim to elucidate the effect that aggregation has on MSCs' ability to suppress T cells within PBMC populations to more fully understand spheroid MSCs' immunomodulatory phenotype. Insight into these phenotypic changes can be used to inform the logical design and application of localized MSC therapies, alone or in combination with drugs for the treatment of inflammatory conditions.

## Materials and Methods

### MSC Spheroid Culture

In this study, both human bone marrow and umbilical cord derived MSCs were used from a total of six donors. Human bone marrow-derived MSCs characterized and obtained from RoosterBio (MSC-001 Lot: 00082, MSC-003 Lot: 00055, and MSC-003 Lot: 00022) were used in addition to a donor obtained from Texas A&M Health Science Center College of Medicine Institute for Regenerative Medicine at Scott & White through a grant from the Office of the Director of the NIH, Grant P40OD011050, resource ID SCR_005522 (Donor # 7083). Umbilical cord-derived MSCs were isolated and characterized in house (Supplemental Figure 1). MSCs were cultured in MEM-α (Fisher Scientific, Cat # BW12-169F) supplemented with 15% premium grade ISIA traceable fetal bovine serum (VWR, Cat# 97068-085, Lot# 059B18), 1% L-glutamine (Life Technologies, Cat # 25030081), and 1% penicillin-streptomycin (Life Technologies, Cat # 15140122). Cells were seeded at a density of 4,000–6,000 cells/cm^2^ and grown until 70–90% confluent. For experiments, 2D-adherent MSCs (Adh) were plated at 5,300 MSCs/cm^2^ in T-75 flasks and incubated at 37°C for 3 days. Adherent MSCs were dissociated with accutase, counted, and plated at the start of each experiment to ensure the starting number of MSCs was comparable in both adherent and spheroid conditions.MSC spheroids (Sph) were formed using a hanging droplet technique as previously described by Ylostalo et al. (31). Briefly, MSCs were resuspended to 1 million cells/mL, and 20 μL droplets of cell suspension were plated on petri dish lids. Sacrificial droplets of media were placed around the cell droplets, and PBS was added to the base of the petri dish to minimize evaporative loss during the spheroid formation process. Lids were inverted and incubated at 37°C for 3 days. A detailed description of the umbilical cord isolation protocol is provided in the Supplemental Materials and Methods.

Adherent and spheroid MSC production of immunomodulatory factors such as IDO, COX-2, CD73, and TGF-β were analyzed at the RNA, protein, or enzymatic product levels. Detailed protocols on RT-PCR, western blot, immunohistochemistry, ELISA, and enzyme activity assays are provided in the Supplemental Materials and Methods.

### PBMC and T Cell Co-culture With Sph and Adh MSCs

Isolated PBMCs or T cells, as described in the Supplemental Materials and Methods, were stained with CSFE (Biolegend, Cat # 423801) as described by the manufacturer. Briefly, PBMCs or T cells were thawed and placed in warm complete RPMI containing 10% premium grade ISIA traceable FBS, 1% penicillin/streptomycin, and 1% L-glutamine for at least 1 h prior to the staining process. Cells were counted and resuspended in PBS at 1 million cells/mL. Two microliter of 5 mM CSFE in DMSO per 10 million PBMC or T cells was vortexed into the cell suspension to obtain a final dye concentration of 1 μM. The PBMCs or T cells were incubated at 37°C in the dark for 15 min. CFSE was then neutralized with complete RPMI, spun down at 500 g for 5 min, and resuspended in complete RPMI. The cells were then allowed to incubate for 30 min at 37°C. Cells were spun and resuspended at 2 million cells/mL in complete RPMI. PBMCs, or T cells for the unstimulated control were removed prior to human CD3/CD28 Dynabead (Thermo Fisher, Cat # 11132D) addition and adjusted to a final concentration of 1 million cells/mL. 240,000 PBMCs, with or without Dynabeads, were added to their respective wells in a 24-well plate.

Adherent MSCs or spheroids were plated 3 days prior to the co-culture. Adherent cells were harvested, resuspended at 1 million cells/mL, and then 60,000 MSCs were plated (~1:4 MSC:PBMC). Three pre-formed 20,000-cell spheroids were then transferred into the wells to achieve an equal number of spheroid MSCs to adherent MSCs at the start of each experiment unless otherwise noted. Visual confirmation was used to ensure an accurate number of spheroids in each well. After 6 days in co-culture, PBMCs or T cells were collected, and their proliferation was analyzed by flow cytometry. CD3/CD28 Dynabead-stimulated and—unstimulated PBMC controls without MSCs were used to set proliferation gates on every experiment. All negative controls without Dynabead stimulation showed no proliferation. PBMC proliferation is listed either as the percentage of total PBMCs to proliferate or normalized to the stimulated control using the formula: (% Proliferation Sample)/(% Proliferation Positive Control). Additional PBMC generational analysis was performed using the FlowJo v10 software and normalized proliferation is described in the Supplemental Materials and Methods. While fetal serum is typically absent C3 complement (32), we validated that heat inactivation was not necessary for our specific lot of FBS in MSC:PBMC co-culture assays. To validate the use of FBS for MSC-PBMC co-culture, PBMCs stimulated with Dynabeads were cultured with or without either 60,000 adherent or spheroid MSCs. This experiment was done in RPMI containing 10% of either normal or heat-inactivated FBS from the same lot (Supplemental Figure 2). No difference was observed with heat inactivation, so all other experiments were done without heat inactivation. Description of the heat-inactivation of FBS is given in the Supplemental Materials and Methods.

### MSC Co-culture in Trans-wells

Adherent and spheroid MSCs were plated into the bottom of 24-well plates in 700 μL of complete RPMI (formulated as described above). PBMCs were stained with CSFE and combined with CD3/CD28 Dynabeads to yield a final concentration of 1 million PBMCs/mL. Trans-well inserts with 3 μm pores (Corning, Cat # 07-200-148) were added to MSC loaded wells and 250 μL of Dynabead-PBMC solution was placed in the inserts. Cells were then cultured for 6 days prior to analysis by flow cytometry as described above.

### PBMC Proliferation With BUD, PGE2, EP2/4 Inhibitors

PBMCs isolated from three independent de-identified blood donors were thawed from a cryo-stock. After recovery, cells were stained with CFSE as described above and activated with CD3/CD28 Dynabeads. 240,000 activated PBMCs with Dynabeads were plated in each experimental well. Each PBMC donor was plated in duplicate with non-treated activated controls on each plate (one plate to determine synergistic effect of budesonide and PGE2 on preventing PBMC activation; one plate used to determine the ability of EP Receptor inhibitors to prevent the synergistic effect). The final working concentrations of all tested compounds were as follows: Budesonide (10 μM), PGE2 (1 μM, Tocris, Cat # 2296), TG4-155 (10 μM, Tocris, Cat # 5052), and L-161,982 (10 μM, VWR, Cat # 75844-788). The same concentrations were used for experiments with and without MSCs.

### Statistics

All statistics and graphs were prepared in GraphPad Prism 8. Prior to statistical analysis, all data were analyzed for normality using a Shapiro-Wilk test. Each group in each data set passed the normality test (*p*-value cut-off of 0.05). Error bars on graphs are represented as mean ± SEM (standard error of the mean) unless otherwise stated in the figure legends. Specific statistical tests and corrections for multiple comparisons are described in the figure legends, and *p* < 0.05 was considered significant for all experiments.

## Results

### Spheroids Cannot Suppress Activated PBMC Proliferation

**Figure 1:**
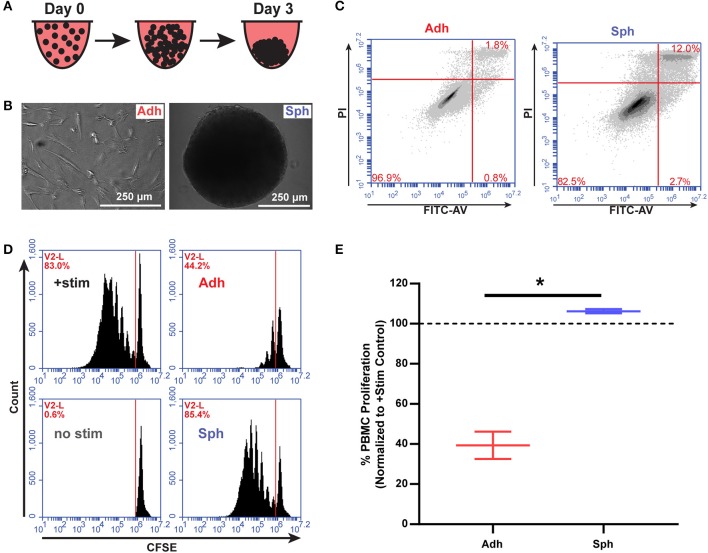
MSC spheroids do not suppress activated PBMCs. **(A)** Schematic of spheroid formation. 20,000 MSCs in hanging droplets form spheroid structures within 3 days of culture. **(B)** Schematic of MSCs in immunomodulatory potency assays. **(C)** Representative flow plots of adherent (Adh) and spheroid (Sph) MSCs stained with FITC-Annexin V and propidium iodide (PI) after 3 days. **(D)** Representative flow cytometry plots of CSFE stained PBMCs with adherent (Adh) or spheroid (Sph) MSCs. **(E)** Quantification of % proliferated PBMCs for *N* = 4 independent MSC donors. Groups in D were analyzed with *t*-test with Welch's correction for unequal standard deviations. All data are represented as mean ± SEM. *p < 0.05.

In order to test the immunomodulatory properties of aggregated MSCs, we used a previously developed protocol (14) to aggregate MSCs into spheroids over 3 days *in vitro* prior to co-culture with human immune cells (Figures 1A,B). We then tested viability of the MSCs and found, consistent with previous reports (18, 33, 34), an increase in Annexin V+/PI+ MSCs in the spheroid group compared to the adherent MSC group (Figure 1C, Supplemental Figure 4). To determine if the increase in cell death results in a reduction in spheroid cell number over time, we used a spheroid optimized assay (CellTiter-Glo 3D, Promega) to quantify the number of viable MSCs in each spheroid compared to a standard curve of viable MSCs. After 4 days in culture, spheroids were found to have the same number of viable MSCs as had originally been seeded (20, 120 ± 860 MSCs/spheroid). Thus, MSC spheroids were neither atrophying by cell death nor growing via proliferation, but remaining at a stable cell number. Next, we sought to understand if aggregation of MSCs altered their interaction with activated T cells. Adherent MSCs are well-known to suppress the proliferation of PBMCs activated with CD3/CD28 Dynabeads or through MLRs. Because of known variability between MSC donors, we used different MSC donors, derived from commercial bone marrow sources and umbilical cords isolated and characterized in house (Supplemental Figure 1). As expected, adherent MSCs showed a robust ability to suppress PBMC proliferation (Figures 1D,E). Surprisingly, aggregation into spheroids completely eliminated their immunosuppressive potency, despite having the same initial number of MSCs used in both adherent and spheroid conditions (Figures 1D,E).

### Spheroid Dose and Contact Factors Are Not Responsible for the Loss of PBMC Proliferation Suppression

**Figure 2:**
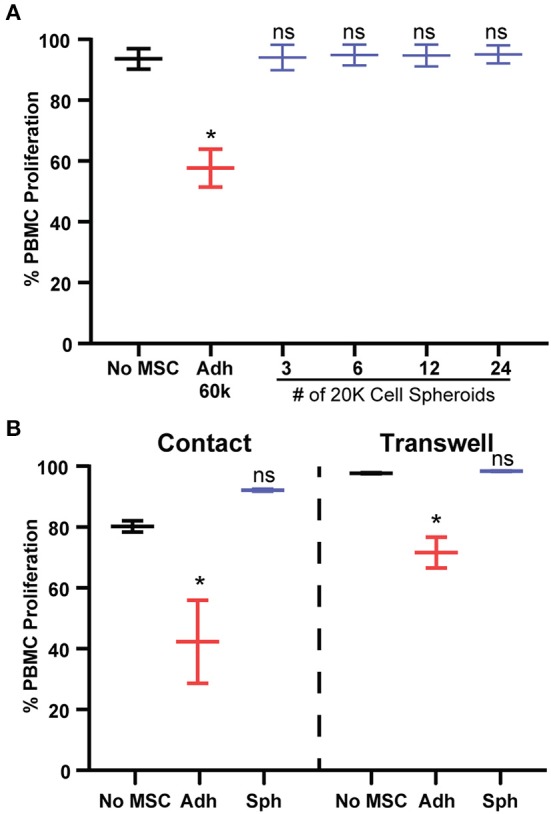
Spheroid dose and contact factors do not explain lack of PBMC suppression. **(A)** Percentage of proliferation of activated PBMCs with escalating doses of 20,000 cell MSC spheroids. One-way ANOVA with Dunnett's multiple comparison's test. **(B)** PBMC proliferation in response to co-culture with adherent or spheroid MSCs at a 1:4 MSC to PBMC ratio with MSCs and PBMCs in direct contact or separated by a trans-well. Two-way ANOVA with Tukey correction for multiple comparisons. All data are represented as mean ± SEM with *p < 0.05.

The dramatic loss in potency we observed just 3 days after spheroid formation was unexpected, so we sought to understand why spheroid MSCs lose immunosuppressive potency and whether it was specific to PBMC suppression. Previous studies have shown that spheroid MSCs display anti-inflammatory actions toward immortalized or mouse macrophages, despite the reduction in cell viability (18, 34–36). In agreement, we found that in co-cultures with primary human macrophages, MSC spheroids enhanced production of anti-inflammatory markers in M2c M-CSF/BUD-polarized macrophages (Supplemental Figures 3B,D), while MSC spheroids in direct contact with M1 LPS/IFN-γ-polarized primary human macrophages suppressed markers of M1 macrophages (Supplemental Figures 3A,C). Thus, spheroid MSCs retain an immunosuppressive activity toward monocyte subsets. Next, we tested the hypothesis that the loss in potency toward PBMC suppression was simply an artifact due to a reduction in the number of MSCs in the spheroids, either due to increased rates of cell death or reduced rates of proliferation. To test this hypothesis, we cultured PBMCs with escalating doses of spheroid MSCs from our original dose of 1:4 (60,000 MSCs:240,000 PBMCs) to a dose 8 × higher of 2:1 (480,000 MSCs:240,000 PBMCs). Regardless of initial dose, spheroid MSCs completely failed to suppress PBMC proliferation (Figure 2A).

An alternative explanation was that spheroid MSCs were being eliminated by contact with cytotoxic T cells. As cytotoxic T cell killing depends on cell contact, we sought to determine if contact factors were responsible for spheroid MSCs loss of PBMC suppression. We used a trans-well assay to prevent direct contact while allowing for exchange of secreted factors between PBMCs and spheroid MSCs. Despite the separation of cells, spheroid MSCs still failed to suppress PBMC proliferation while adherent MSCs remained suppressive (Figure 2B).

### Spheroid MSCs Produce Immunomodulatory Factors

**Figure 3:**
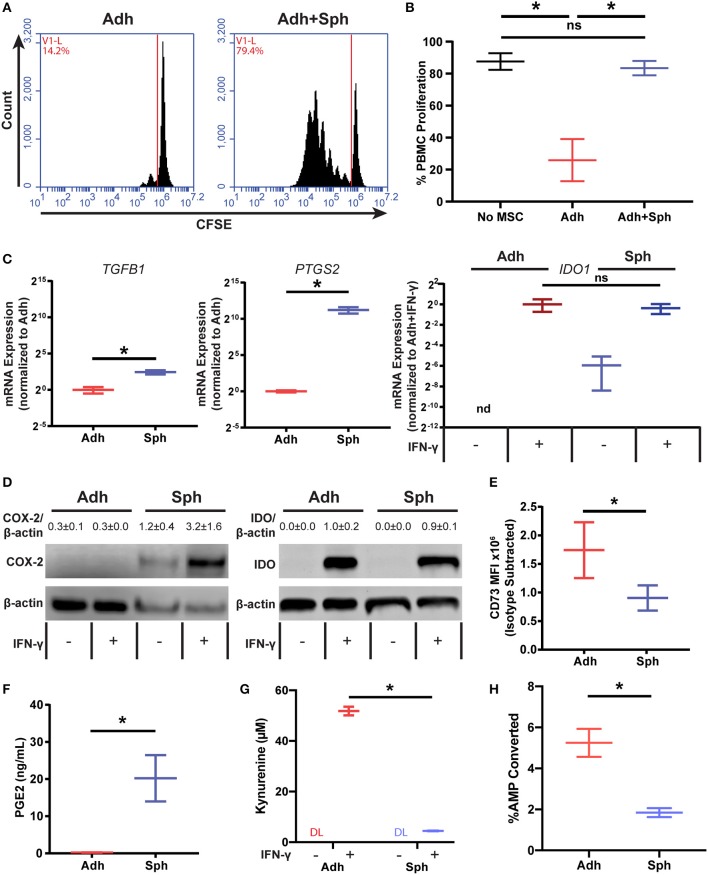
Spheroid MSCs display an altered immunomodulatory profile. Representative flow cytometry plots of activated PBMC co-cultures with adherent MSCs or adherent MSCs with spheroids added in at a 1:5 adherent to spheroid ratio **(A)** with quantification of % proliferation in **(B)**. Data was analyzed using a one-way ANOVA with Tukey correction for multiple comparisons. **(C)** RT-PCR gene expression data for *TGFB1, PTGS2*, and *IDO1* in adherent and spheroid MSCs alone. MSCs used for *IDO1* RNA were stimulated with 100 ng/mL IFN-γ for 3 days prior to analysis. RNA data for *TGFB1* and *PTGS2* was analyzed with a *t*-test with Welch's correction for unequal standard deviations. **(D)** Protein quantification by western blot of COX-2 and IDO for adherent and spheroid MSCs with and without IFN-γ treatment. Reported protein ratios were averaged across *N* = 3 independent experiments. **(E)** Flow cytometry analysis of CD73 expression on adherent and spheroid MSCs. Statistics were performed using a *t*-test with Welch's correction for unequal standard deviations. PGE synthase, IDO, and CD73 enzyme products PGE2 **(F)**, kynurenine **(G)**, and free phosphate **(H)**, respectively, measured for spheroid and adherent MSCs after 3 days in culture (60,000 cells per condition). Statistical analysis in **(F–H)** were performed using a *t*-test with Welch's correction for unequal standard deviations. All data are represented as mean ± SEM with *p < 0.05 (DL, Below Detection Limit of Assay).

Finding that the loss of potency could not be attributed to a reduction in the number of cells or cell contact mediated factors, we next wanted to determine if spheroid MSCs were actually promoting, rather than inhibiting, PBMC proliferation. To test this, we cultured CD3/CD28 Dynabead-activated PBMCs with either adherent MSCs, alone or a combination of adherent and spheroid MSCs (1:5 ratio of Adh to Sph MSCs). As expected, adherent MSCs alone were suppressive, but when spheroids were added to adherent co-cultures, PBMC proliferation significantly increased (Figures 3A,B). Thus, spheroid MSCs appear to either actively support PBMC proliferation or potentially inhibit suppression by adherent MSCs.

We next wanted to determine if spheroid MSCs produced the immunosuppressive factors commonly associated with MSCs. Adherent and spheroid MSCs were analyzed for gene expression and production of known immunomodulatory mediators (Figure 3C). We measured mRNA levels of *TGFB1* encoding for TGF-β, *IDO1* encoding for indolamine 2,3-deoxgenase, and *PTGS2* encoding for COX-2, which is a critical enzyme in the prostaglandin synthesis pathway (Figure 3C). Since IDO is an inducible protein, we treated MSCs with or without rhIFN-γ to turn on IDO transcription. We found that *TGFB1*and *PTGS2* transcripts were increased 2- and 500-fold, respectively, in spheroids compared to their adherent counterparts. With rhIFN-γ stimulation, *IDO1* was expressed at a similar level in both adherent and spheroid MSCs. However, while *IDO1* was not detectable in adherent MSCs without rhIFN-γ stimulation, spheroid MSCs showed small but detectable *IDO1* expression even without rhIFN-γ stimulation.

With both *PTGS2* and *IDO1* showing differences at the RNA level, we next measured the abundance of the enzymes they encode, COX-2 and IDO, respectively (Figure 3D). COX-2 protein was much greater in spheroid compared to adherent MSCs, while IDO expression for both rhIFN-γ-stimulated conditions were at similar levels, consistent with mRNA measurements. However, in both groups without rhIFN-γ, there was no IDO protein detected. Additionally, we examined CD73 (ecto-5′-nucleotidase), a surface marker for hMSCs that also has immunomodulatory function through the conversion of AMP to adenosine and free phosphate. Since CD73 is expressed by all MSCs (Supplemental Figure 1), we looked at changes in surface protein levels of the enzyme by flow cytometry. Unlike IDO, which was unchanged, and COX-2, which was elevated, surface expression of CD73 was significantly lower in spheroid compared to adherent MSCs (Figure 3E).

We next measured the activity of the three immunomodulatory enzymes—COX-2, IDO, and CD73—in spheroid MSCs. While PGE2, the product of COX-2 and prostaglandin E synthase, was produced by adherent MSCs, it was ~100-fold higher in spheroid compared to adherent MSCs (Figure 3F). In contrast, the levels of enzymatic products of IDO and CD73, kynurenine and free phosphate, were 10- and 3-fold lower, respectively, in spheroid MSC cultures (Figures 3G,H). While expected for CD73, since surface expression was reduced in spheroids, these results showed an unexpected disconnect between levels of IDO protein and the activity of the enzyme. Thus, spheroid MSCs loss of PBMC suppressive potency is associated with a dramatic increase in the production of PGE2 and a significant reduction of activity in both IDO and CD73, revealing a distinct immunomodulatory phenotype of spheroid MSC.

### Spheroid MSC Suppression of PBMC Proliferation Can Be Partly Restored in the Presence of Budesonide

**Figure 4:**
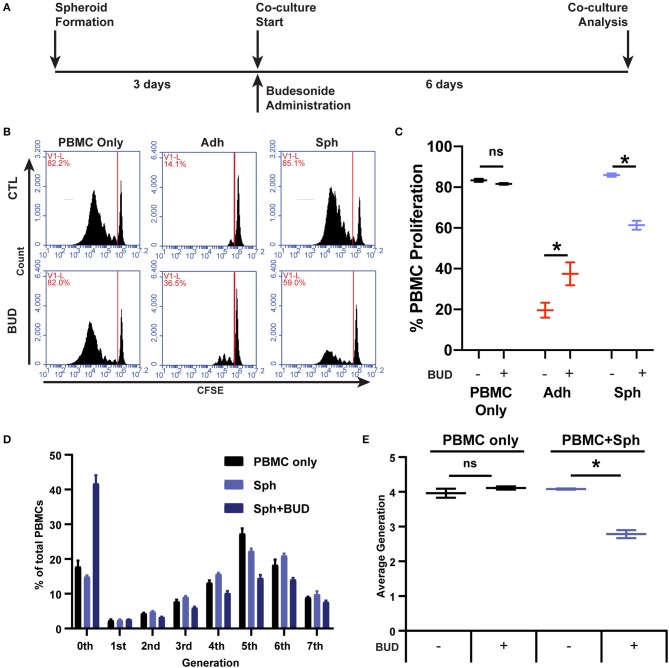
Budesonide synergizes with spheroids to suppress PBMC proliferation. **(A)** Timeline schematic for spheroid co-culture with budesonide administration. Representative flow cytometry plots **(B)** for PBMC co-cultures with and without 10 μM budesonide treatment, with quantification of % PBMC proliferation **(C)**. **(D)** PBMC generational analysis using FlowJo v10 proliferation software from flow cytometry data collected in **(B,C)**. Percentage of total PBMCs is displayed for each generation (8-peak model). **(E)** Average generation of PBMCs was calculated from D, as described in the Supplemental Materials and Methods. Two-way ANOVA with Sidak correction for multiple comparisons between control and budesonide groups. All data are represented as mean ± SEM with *p < 0.05.

While loss of PBMC suppression is concerning, in clinical translational settings, MSCs are often not administered alone but in combination with standard-of-care therapies. For inflammatory conditions like GvHD, Crohn's disease, and ulcerative colitis, locally administered glucocorticoid steroids can be preferred for treatment of lesions (37). Budesonide is a commonly used glucocorticoid steroid for local applications due to its poor systemic absorption and high first-pass metabolism (37). Thus, we wanted to determine if spheroid MSC interactions with PBMCs would be influenced by the presence of budesonide (Figure 4A). We found that spheroids or budesonide (10 μM) alone were not capable of suppressing CD3/CD28-stimulated PBMC proliferation, but the combination of spheroids with budesonide worked synergistically to significantly suppress PBMC proliferation (Figures 4B,C). When we tested the effect of soluble budesonide with adherent MSCs, we found that there was no benefit; rather, we observed a small decrease in MSC's ability to suppress PBMC proliferation (Figures 4B,C).

In addition to proliferation, we examined whether this synergy affected secretion of effector cytokines produced by PBMCs in co-culture with spheroid MSCs, specifically IFN-γ, IL-10, and granzyme B. Spheroid MSCs alone had no effect on the levels of IFN-γ or granzyme B in the co-culture, but there was a significant decline in IL-10 (Supplemental Figure 5). Budesonide alone had a significant suppressive effect on PBMC production of both IFN-γ and granzyme B (Supplemental Figures 5A,C), but no significant effect on IL-10 secretion (Supplemental Figure 5B). Together, budesonide and spheroid MSCs led to an even greater reduction in IFN-γ, IL-10, and granzyme B levels (Supplemental Figures 5A–C). Thus, budesonide synergy with MSC spheroids affects both PBMC proliferation as well as the resultant cytokine environment.

While suppression was enhanced with the combination of budesonide with spheroid MSCs, the suppressive profile of spheroid MSCs with budesonide was qualitatively different from that of adherent MSCs. From analysis of the CFSE generation peaks, we found the proportion of proliferated cells within each generation was similar between the spheroid MSC:PBMC co-cultures with and without budesonide (Figure 4D). However, the proportion of non-proliferative to proliferative cells, regardless of generation, was shifted to the non-proliferative gate when budesonide was added with spheroid MSCs yielding an overall lower average generation (Figures 4D,E). This suppression profile was surprising, as adherent MSCs typically halt proliferation after 0–2 doubling events (Figure 1C). Thus, unlike adherent MSCs, spheroid MSCs in combination with budesonide display a distinct pattern of suppression. As this suppression profile was distinct from adherent MSCs, we hypothesized that it was not driven by kynurenine. To test this hypothesis, we treated spheroid MSCs with and without budesonide in the presence of the inflammatory cytokine IFN-γ to determine if budesonide increased spheroid MSC production of kynurenine. We found that budesonide had no effect on spheroid MSC production of kynurenine, suggesting there is likely another suppressive factor dominating the synergistic effect seen with spheroid MSCs and budesonide in co-culture (Supplemental Figure 6).

### Spheroid Produced PGE2 Is Not Sufficient to Suppress PBMC Proliferation

**Figure 5:**
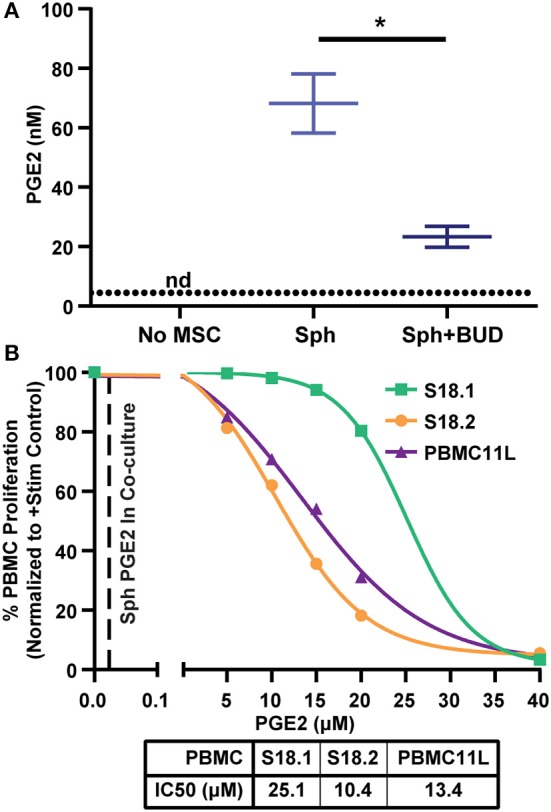
Spheroid produced PGE2 levels alone are not sufficient to suppress PBMCs. **(A)** PGE2 measured by competitive ELISA in PBMC cultures without MSCs or co-cultures with spheroid MSCs with and without 10 μM budesonide (*t*-test with Welch's correction for unequal standard deviations. All data are represented as mean ± SEM with *p < 0.05). Dotted line represents the assay detection limit. **(B)** Escalating doses of synthetic PGE2 was added to activated PBMCs and proliferation was measured to determine the IC50 values of three independent PBMC donors. Replicates for each PBMC donor were averaged and the mean is displayed. Each curve represents one of three different PBMC donors used for this experiment (*N* = 3 PBMC donors). IC50 values were calculated from a four-parameter log (inhibitor) vs. response model in GraphPad Prism 8 for each PBMC donor independently. Dashed line represents average PGE2 concentration from spheroid with budesonide group in **(A)**.

Because PGE2 was the most highly up-regulated factor we observed (Figure 3), we wanted to determine if PGE2 alone could induce suppression of PBMC proliferation. Therefore, we measured PGE2 in MSC-PBMC co-cultures and found that PGE2 was elevated in co-cultures containing spheroids reaching a peak of 85 nM (Figure 5A). As expected (38), PGE2 levels were higher without budesonide, but PGE2 from both spheroid MSC groups were far higher than the levels measured in the stimulated PBMC controls (Figure 5A). To determine the IC50 of PGE2 required to suppress PBMCs by itself, we treated activated PBMCs with escalating doses of synthetic PGE2 and measured proliferation of the cells. The IC50 values calculated for three different PBMC donors were in the range of 10–25 μM, which is over 100X higher than the peak PGE2 concentration measured in co-cultures with spheroid MSCs (Figure 5B). As the levels of PGE2 produced from MSC spheroids were far too low to account for PBMC suppression by itself, we theorized spheroid-produced PGE2 might be synergizing with budesonide to yield a suppressive effect.

### Spheroid MSCs Secrete PGE2 That Synergizes With Budesonide to Suppress Activated T Cell Proliferation

**Figure 6:**
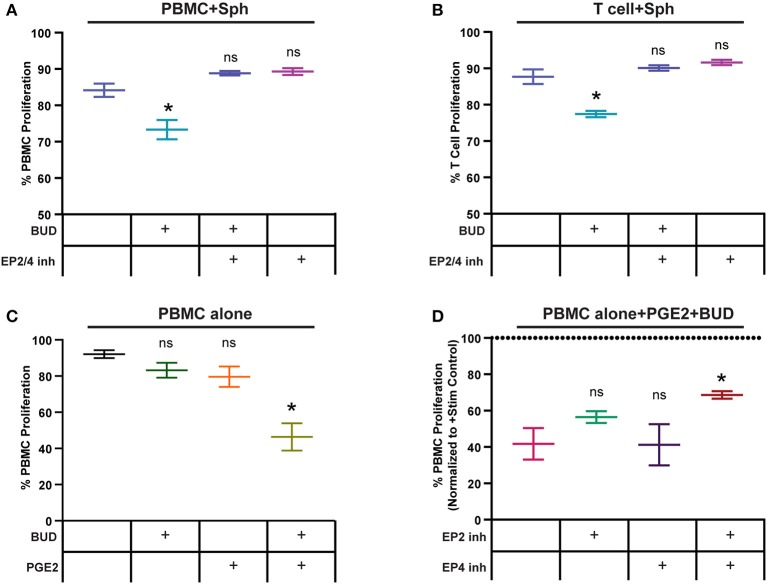
Spheroid PGE2 synergizes with budesonide via EP2/4 receptors on T cells. **(A)** PBMC or **(B)** T cell proliferation response to spheroids, with or without 10 μM budesonide or EP2/4 inhibitors TG4-155/L161, 982 (10 μM). Statistical analysis was done using one-way ANOVA with Sidak correction for multiple comparisons to the untreated no drug group. **(C)** PBMC proliferation alone or after exposure to 1 μM synthetic PGE2 and 10 μM budesonide. Statistical analysis was done using one-way ANOVA with Sidak correction for multiple comparisons to the untreated PBMC alone group. **(D)** PBMCs treated with budesonide and synthetic PGE2 as in **(C)** with or without EP2 and EP4 inhibitors TG4-155 and L-161, 982 at 10 μM. Statistical analysis was done using one-way ANOVA with Sidak correction for multiple comparisons to the control group. All data are represented as mean ± SEM with *p < 0.05.

We wanted to determine if the synergy between PGE2 from spheroid MSCs and budesonide in our PBMC potency assay could be broken by blocking PGE2 signaling. To test this, we treated PBMCs with MSC spheroids and budesonide and added in selective antagonists for PGE2 receptors EP2 and EP4 (TG4-155 and L-161,432), which have been implicated as critical for PGE2-mediated suppression of PBMCs (39). As before, spheroids and budesonide synergized to suppress PBMC proliferation (Figure 6A). However, when the EP2/4 inhibitors were added, this suppressive effect was lost. As none of the inhibitors or budesonide combinations impacted PBMC proliferation alone (Supplemental Figure 7A), these results suggest that synergy between spheroid MSCs and budesonide is due at least in part to signaling by PGE2 mediated through EP2/EP4 receptors.

We next wanted to determine if the synergy observed between spheroid MSCs and budesonide could act directly on T cells or if the suppressive effect was dependent on non-T cell bystanders within the PBMC population. We used a negative selection kit to remove non-T cell populations from PBMCs and co-cultured spheroid MSCs with the remaining T cells. Addition of spheroids and budesonide to CD3/CD28 Dynabead activated T cells resulted again in a suppressive effect on proliferation, which was blocked by addition of EP2/4 inhibitors (Figure 6B). As with PBMCs, drug combinations alone did not have any significant impact on T cell proliferation (Supplemental Figure 7B). These data suggests budesonide and spheroid MSC-produced PGE2 can act on T cells without requiring a non-T cell bystander to mediate the suppressive effect.

### Budesonide Works Synergistically With PGE2 to Suppress PBMC Proliferation

Since budesonide can act on both the MSCs and PBMCs, we next wanted to determine if synthetic PGE2 and budesonide alone could work synergistically on PBMCs to replicate the suppression seen with spheroid MSCs, to support the hypothesis that PGE2 from spheroid MSCs and budesonide work synergistically to suppress PBMC proliferation. We treated PBMCs with a low dose of PGE2 with or without budesonide. As hypothesized, neither PGE2 or budesonide alone suppressed PBMC proliferation while the combination led to a large and significant reduction in proliferation (Figure 6C). As with co-cultures with MSC spheroids, addition of EP2/EP4 inhibitors to PBMCs treated with synthetic PGE2 and budesonide resulted in loss of suppression and increased PBMC proliferation (Figure 6D). Blockade of EP2 alone only partially blocked the suppressive effect, while inclusion of both inhibitors together led to a more substantial decrease in the suppression of PBMC proliferation.

### Both Bone Marrow and Umbilical Cord Derived MSC Spheroids Lose Suppression, but Regain Some Suppression With Budesonide Treatment for Multiple PBMC Donor Pairings

**Figure 7:**
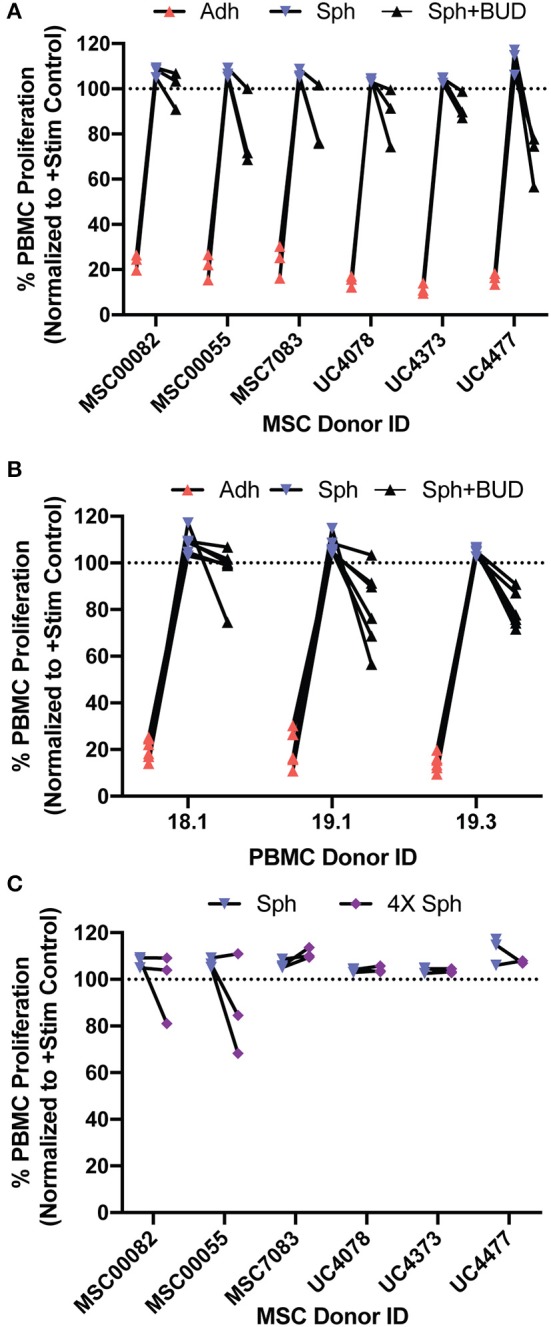
Spheroid MSC loss of suppression and synergy with budesonide is generalizable across multiple bone marrow and umbilical cord derived MSC-PBMC pairings. Six MSC donors (MSC00082, MSC00055, MSC7083, UC4078, UC4373, and UC4477) were co-cultured with three independent PBMC donors (18.1, 19.1, 19.3) to create 18 unique MSC-PBMC pairings (*N* = 6 MSC donors, *N* = 3 PBMC donors). **(A)** PBMC proliferation for adherent, spheroid, or spheroid with 10 μM budesonide was analyzed, normalized to the stimulated PBMC controls, after 6 days. **(B)** Data collected in A, grouped by PBMC donor. **(C)** 240,000MSCs in 20,000 cell spheroids, 4× the cell number in the spheroid group were cultured with PBMCs and proliferation of the PBMCs was measured.

In this work, we had two main findings regarding spheroid MSCs: that they lose suppression of PBMC proliferation and that treatment with budesonide can partially restore spheroid MSC potency. Since there is often high variability in MSC sources, we wanted to determine if these findings were generalizable across a range of MSC donors and multiple PBMC donors. To test the generalizability of our findings, we generated 18 unique MSC:PBMC pairings using bone marrow MSCs from three donors and umbilical cord MSCs from three donors paired with three different PBMC donors and then examined PBMC suppression of adherent MSCs, spheroid MSCs, or spheroid MSCs with budesonide for each pairing. Disaggregating the data by both MSC donors (Figure 7A) and PBMC donors (Figure 7B) revealed that adherent MSCs in all 18 pairings suppressed PBMC proliferation while none of the spheroid MSC containing conditions displayed any suppression of proliferation (Figure 7A). Thus, the observed loss of PBMC suppression by spheroid MSCs was generalizable across all 18 donor pairings. The addition of budesonide to spheroid MSC co-cultures reduced PBMC proliferation in all 18 donor pairings compared to spheroid MSC groups and resulted in a suppressive phenotype in 14 of the 18 pairings (Figure 7A), suggesting that rescue of spheroid MSC-suppressive phenotype is broadly generalizable.

Finally, we tested whether increasing the dose of MSCs by 4X to account for differences in MSC number due to differences in viability or proliferation would lead to suppression of PBMC proliferation. In agreement with our original observation in Figure 2A, a two-way ANOVA revealed that increasing the dose of spheroid MSCs (from 3 spheroids to 12 spheroids) did not significantly change the level of PBMC suppression (Figure 7C). Thus, increasing the spheroid MSC dose is not a reliable strategy to improve spheroid MSC suppression of PBMC proliferation.

## Discussion

MSC immunomodulatory phenotype has been studied for years using 2D-adherent culture on tissue culture plastic; however, MSCs administered *in vivo* experience a very different environment. Specifically, there is evidence that in particular local delivery contexts, MSCs aggregate *in vivo* (14, 17), which alters the repertoire of trophic factors the cells produce (18, 19, 40). This raises the question: Are spheroid MSCs more immunomodulatory potent than their adherent counterparts, or do they have a different immunomodulatory profile?

Suppression of PBMCs, T cells, or MLRs have long been used as *in vitro* assays to assess the potency of human MSCs prior to *in vivo* use (3, 27, 28, 41). In this study, we examined adherent and spheroid MSC potency in a series of *in vitro* assays using exclusively primary human cells. We expected spheroid MSCs to suppress activated PBMC proliferation in a dose-dependent manner but were surprised to find spheroid MSCs displayed no suppressive potency at all, as measured by PBMC proliferation, IFN-γ, and granzyme B assays.

This complete and rapid loss of potency was unexpected and initially thought to be an artifact of our potency assays or choice of donors. However, the same loss in potency upon spheroid formation was seen with six independent human MSC donors, both umbilical cord and bone marrow derived, paired with three separate PBMC donors. When the multi-donor data were analyzed by two-way ANOVA with Tukey's multiple comparisons test, it showed spheroid MSCs were significantly worse than adherent MSCs (95% CI of difference: −92 to −85) at suppressing PBMC proliferation and that addition of budesonide resulted in a significant improvement in spheroid MSC suppression (95% CI of difference: 12 to 31). Overall, 95% of the variation observed was attributed to the co-culture condition, while the PBMC donor, MSC donor, and MSC donor:PBMC donor interaction each attributed to <1% of the observed variation. This demonstrated that the loss in potency was not dependent on the donor pairing used.

These finding could seem to be in contrast to work done by Zimmermann et al., which showed that untreated spheroid MSCs suppressed activated PBMCs at 3:1 MSC:PBMC ratio (42). However, the size of the spheroids studied are critically different. While we used 20,000 MSCs in all of our spheroids, Zimmermann et al. used spheroids containing only 500 cells, and aggregate size has been shown to substantially affect spheroid secretome (19, 42). While such small sizes are controllable in the lab, we chose larger aggregates to more closely approximate spontaneous aggregation that would occur upon local injection of a concentrated cell suspension *in vivo* (14).

Follow up analysis of the expression and production of immunomodulatory factors showed an overall shift in the immunomodulatory phenotype between adherent and spheroid MSCs. While MSCs are known for their anti-inflammatory properties, some of these immunomodulatory factors are not upregulated until MSCs are exposed to inflammatory factors, including IFN-γ, which has been shown to upregulate IDO and COX-2 protein expression. We used IFN-γ to induce expression of both IDO and COX-2 in order to characterize differences between adherent and spheroid MSC responses to a controlled inflammatory stimulus. Expression of *TGFB1*, protein levels of CD73, and activity of both IDO and CD73 were different between adherent and spheroid MSCs. The most striking observed change in spheroids, however, was the increase in *PTGS2* expression, COX-2 protein, and PGE2 secretion in spheroid MSCs compared to their adherent counterparts.

Not only were spheroids not able to suppress T cell proliferation, but they actually supported proliferation, likely due to a shift in balance between production of anti- versus pro-inflammatory factors. While best known for their anti-inflammatory properties, MSC constitutively express an array of classically pro-inflammatory cytokines and chemokines (43). In addition, specific environmental factors, such as LPS (44), TNF-α (43, 45), and palmitate (27, 44), have been shown to polarize MSCs toward a pro-inflammatory state. In spheroids, Bartosh et al. have shown MSCs also produce pro-inflammatory cytokines, such as IL-1α/β (14), which can promote T cell expansion (46). While not addressed in this study, more research is needed to understand the mechanism causing spheroid MSCs to lose the ability to suppress activated PBMCs and how combinations of different stimuli control the balance of anti-inflammatory and pro-inflammatory products produced by MSCs.

Other stimuli that affect MSC interactions with immune cells are the standard-of-care immunosuppressants patients receive prior to MSC therapy. Glucocorticoid steroids are commonly used on patients with inflammatory diseases and can impact both the recipient's immune cells as well as the transplanted MSCs. Studies of steroids' effects on MSCs have been limited but suggest this interaction requires more extensive investigation and understanding. For example, Chen et al. showed that mouse MSCs treated with dexamethasone decreased their suppression of anti-CD3 activated splenocytes (47). When they examined human MSCs, they found dexamethasone reduced MSC expression of inflammation-inducible immunomodulatory proteins. However, there is evidence that steroid treatment may have a beneficial effect on MSC function as well. Dexamethasone treated MSCs suppress IFN-γ, perforin, and CD69 expression in NK cell populations (48). In addition, we have previously shown that loading MSCs with intracellular microparticles containing budesonide enhances their suppression of peripheral blood mononuclear cells (PBMCs) (2). However, in the current study, adherent MSCs treated with solubilized budesonide in co-culture with PBMCs did not demonstrate improved suppressive capability. In this study, we used budesonide, a commonly used steroid for local administration, to understand glucocorticoid's impact on spheroid MSC:PBMC interactions. To our surprise, while neither spheroid MSCs nor budesonide suppressed T cell proliferation alone, together they significantly suppressed T cell proliferation. Interestingly, 3 of the 4 MSC-PBMC pairings, which did not show this suppression between spheroid MSCs and budesonide, were with PBMC donor 18.1, which we had previously observed was less sensitive to PGE2 than 2 other PBMC donors (Figure 5B). This suggests that differences in PBMC sensitivity to PGE2 may influence the ability of budesonide to synergize with spheroid PGE2 to suppress PBMC proliferation. While many spheroid MSC-based factors could synergize with steroids, we focused on understanding the role spheroid produced PGE2, the most highly upregulated factor in spheroid MSCs, and played in the observed synergy. PGE2, while present in MSC spheroid:PBMC co-culture media, was at a concentration 100-timestoo low to induce suppression by itself. We found intact PGE2 signaling to be critical for synergy with budesonide, as inhibition of EP2/4 receptors eliminated the synergy entirely. Furthermore, the synergy between PGE2 and budesonide appears to not rely on non-T cell populations, as their depletion left the synergy between spheroid PGE2 and budesonide intact. Thus, synergy appears to act directly on T cells and be reliant on intact EP2/4 signaling. We confirmed that kynurenine production from spheroid MSCs was not enhanced through budesonide treatment, further supporting our data showing that MSC suppression of PBMC proliferation had shifted from a kynurenine dominated mechanism to a PGE2-budesonide-mediated mechanism.

Interestingly, both PGE2 and glucocorticoids have known effects on TCR signaling. PGE2 signaling via EP2/4 increases cAMP levels which activate PKA, which in turn can phosphorylate LCK^505^, which is critical in the initial formation of the TCR/CD3 signaling complex (49). Thus, PGE2 signaling could be enhanced by glucocorticoids as they can bind to cytoplasmic glucocorticoid receptor (GR) and also activate PKA leading to inhibition of LCK (50). This signaling then precipitates disassociation of the LCK/FYN complex, reducing TCR signaling (50). In addition, PGE2 has also been shown to inhibit expression of inflammatory mediators such as IFN-γ and IL-17 via transcriptional regulation (3). The glucocorticoid-GR complex acts as a transcription factor to modulate activation of inflammatory genes, often inhibiting NFκB signaling, which is critical for expression of cytokines upon T cell activation (51).

In our system, we have shown that PGE2 signaling through the EP2/4 receptors is critical for spheroid MSC-budesonide synergy in the suppression of human T cells. With signaling downstream of both PGE2 and budesonide impinging upon the PKA/LCK/TCR pathway, together they appear capable of a suppressive effect that neither can achieve alone. Furthermore, by replacing MSC spheroids with synthetic PGE2, we were able to replicate this synergistic effect, suggesting PGE2 from spheroids is indeed the predominant signaling molecule produced by MSCs that works synergistically with budesonide to suppress T cell proliferation.

While this study provides significant insight into spheroid MSC interaction with T cells, there are limitations which should be considered when interpreting the data. Firstly, all the PBMC suppression work has been performed using immune cells isolated from peripheral blood. However, upon local injection, MSCs would interact with both tissue resident as well as recruited T cells from the periphery. Tissue-resident T cells can have significant differences in responses including gene expression, proliferation, and motility (52). Additionally, our study predominantly used PBMC proliferation response as a measure of PBMC-based inflammation. While proliferation has been shown to correlate with inflammatory cytokine secretion under certain circumstances, proliferation and effector functions including cytokine secretion, cytotoxic killing, and T cell polarization are not always correlated (2, 53). Additionally, this study did not look at other immune subpopulations, which may make up a significant portion of inflammatory mediators in tissues such as NK cells which have both cytolytic and inflammatory function. Further investigation into interactions with other immune cells and complex interactions between multiple immune cell populations is needed to better understand the function of aggregated MSCs post-transplantation *in vivo*.

In conclusion, aggregated human MSCs lose the ability to suppress activated T cell proliferation. While spheroid MSCs do not suppress T cell proliferation alone, when placed in an environment with budesonide, a glucocorticoid steroid, they regain suppressive potency. Using a series of inhibitor and add-back studies, we found spheroid MSC-produced PGE2 can act on EP2/EP4 receptors on T cells in synergy with budesonide to suppress T cell proliferation. We found that these findings were generalizable across all 18 MSC:PBMC donor pairings we tested. While synthetic PGE2 is sufficient to replicate the spheroid-budesonide mediated suppression, this does not mean MSC spheroids are therapeutically unnecessary. The advantage of cell therapy over synthetic drugs has always been their ability to produce a host of factors in a coordinated fashion to resolve inflammation and promote tissue regeneration. In this study, we focused on spheroid MSCs immunomodulatory properties, but did not assess their production of antimicrobial, growth, neuroprotective, or anti-apoptotic factors. Depending on the needs of a specific disease indication, spheroid MSCs may retain a sufficient repertoire of therapeutic mediators to justify their use, and the choice of MSC therapy must always be made within the context of the disease process and required mechanisms of action (54). For inflammatory conditions, suppression of T cells is a critical part of resolving inflammation, and this study highlights the importance of understanding the effect of cell aggregation on MSCs' immunomodulatory phenotype and how that phenotype's efficacy depends on the presence of other environmental cues.

## Data Availability Statement

The datasets generated for this study are available on request to the corresponding author.

## Ethics Statement

Human umbilical cords were collected from the University of Iowa Women's Health Tissue Repository's Maternal Fetal Tissue Bank with written informed consent from the mothers (IRB#200910784).

## Author Contributions

Conception and design of experiments was done by AB, LD, LB, and JA. Collection and/or assembly of data and manuscript writing was done by AB, LD, LB, DB, MS, and JA. Data analysis and interpretation was performed by AB, LD, LB, MS, and JA. Final approval of manuscript was given by AB, LD, LB, DB, MS, DS, and JA. Provision of study material or patients was supplied by DS and JA. Financial support was provided by JA.

### Conflict of Interest

The authors declare that the research was conducted in the absence of any commercial or financial relationships that could be construed as a potential conflict of interest.
