# Supplementary material for: Aggregation of Human Mesenchymal Stromal Cells Eliminates Their Ability to Suppress Human T Cells
Source: Front Immunol. 2020 Feb 25;11:143. doi: 10.3389/fimmu.2020.00143 (PMC7052295; doi:10.3389/fimmu.2020.00143)
Supplement: Supplementary file 1 [file Data_Sheet_1.docx]

## Supplemental Materials and Methods

### MSC Isolation from Umbilical Cords

Human umbilical cords were collected from the University of Iowa Women’s Health Tissue Repository’s Maternal Fetal Tissue Bank with written informed consent from the mothers (IRB#200910784). Tissue samples were de-identified before transport to the lab where MSCs were outgrown from umbilical cord pieces. Briefly, 1 cm pieces were cut cross-sectionally from the umbilical cords, washed in PBS, and cut into smaller pieces. These pieces were placed into dry petri dishes and left to adhere prior to media addition. MEM-α (Invitrogen, Cat # 12561072) supplemented with 15% (v/v) fetal bovine serum (FBS, VWR, Cat # 059B18), 1% (v/v) L-glutamine (Cat # 25030081, Life Technologies), 1% (v/v) penicillin-streptomycin (Life Technologies, Cat # 15140122), and 2% (v/v) Amphotericin B (Sigma Cat # A2942) was added to cover the bottom of the dish and media was changed every 2-3 days for 9 days. Tissue pieces were removed, cells were washed with PBS, and then detached with Accutase (Innovative Cell Technologies, Cat # AT-104). Harvested cells were then plated, further expanded, and frozen down at 1 million cells/mL in CryoStor CS5 freezing media (Sigma, Cat # C2999) at 1°C/min in CoolCell LX freezing containers (Corning, Cat # BCS-405). After cells were cooled for at least 3 hours at -80°C, they were transferred to liquid nitrogen for long term storage. MSC identity was confirmed using tri-lineage differentiation and surface marker assessment as described below. For this study, three umbilical cord donors UC4477, UC4373, and UC4078 were used (Supplemental Figure 1).

Umbilical cord cells were confirmed to meet the minimal MSC criteria for differentiation by differentiating cells into adipocytes, osteocytes, or chondrocytes, using adipogenic (Biological Industries, Cat # 05-330-1B), osteogenic (Biological Industries, Cat # 05-440-1B), or chondrogenic (R&D systems, Cat # SC006) differentiation medias. For adipogenic and osteogenic differentiation, cells were plated in 24-well plates at a cell density of 60,000 cells per well until 80% confluence. Media was exchanged for adipogenic or osteogenic media and the cells were allowed to culture for 14 days with media exchanged every 3 days. Adipogenic lineage was confirmed by fluorescent microscopy after AdipoRed staining for lipid droplets. Osteogenic lineage was confirmed by brightfield imaging after Alizarin Red staining for mineral deposition. For chondrogenic differentiation, MSCs were transferred to a 15 mL conical tube at 250,000 cells per tube. The cells were washed and resuspended in chondrogenic differentiation media, then centrifuged. The pelleted cells were grown for 14 days and media was exchanged every 2-3 days. The pellet was fixed, cryosectioned, and stained with Safranin-O prior to brightfield imaging.

Umbilical cord cells were also confirmed to meet the minimal MSC criteria for surface markers as established by the ISCT by staining for the presence of CD73, CD90, and CD105 and the absence of CD11b, CD19, CD34, CD45, and HLA-DR. PE.Cy7-CD73 (BD Biosciences, Cat # 561258), PE-CD90 (BD Biosciences, Cat # A15794), and FITC-CD105 antibodies (BD Biosciences, Cat # 561443) with corresponding isotype controls (PE.Cy7 Mouse lgG1k (BD Biosciences, Cat # 557872), PE Mouse lgG1 (Invitrogen, Cat # GM4993), and FITC Mouse lgG1k (BD Biosciences, Cat # 556649)) were used to assess positive markers. PE hMSC Negative Cocktail (BD Biosciences, Cat # 562530) was used to assess the negative markers and included appropriate isotype controls. Cells were analyzed on a BD Accuri C6 flow cytometer.

### PBMC and macrophage isolation from leukocyte reduction cones

Leukocyte reduction cones were obtained from the DeGowin Blood Center, University of Iowa Hospital and Clinics. Written informed consent was obtained from each donor. Information was de-identified according to Institution Review Board regulations (IRB#201103721) and the Usage Agreement. The sample was diluted to 50 mL with base RPMI 1640 (Life Technologies, Cat # 11835030). 16 mL of Ficoll (GE Healthcare, Cat # 17-5442-02) was added into 50 mL LeucoSep tubes (Greiner Bio, Cat # E140933Y) and centrifuged at 500g for 1 minute to partition the Ficoll beneath the filter. Half of diluted blood was then transferred into each LeucoSep tube and centrifuged for 30 minutes at 600g with acceleration of 5 and brake off. After spin, the buffy coat which contained peripheral blood mononuclear cells (PBMCs) was collected and washed twice with 10 mL of PBS containing 2% FBS. To remove the remaining red blood cells, the cell pellet was resuspended with 5 mL of RBC Lysis Buffer (Biolegend, Cat # 420301) diluted to 1x in water and incubated on ice for 5 minutes. The suspension was diluted to 30 mL with PBS containing 2% (v/v) FBS, centrifuged at 500g for 5 minutes, and resuspended with 30 mL of complete RPMI containing 10% FBS, 1% L-glutamine, and 1% penicillin-streptomycin.

To cryopreserve the PBMCs, PBMCs were resuspended at 20 million cells/mL in complete RPMI. A 2X freezing solution was prepared made of 20% DMSO and 80% FBS. This freezing solution was then mixed 1:1 with the cells to obtain a 10 million cell/mL solution containing 10% DMSO and 40% FBS. Cells were then immediately aliquoted into 2mL cryo-tubes and placed in a CoolCell (Biocision, Cat # BCS-405). Cells were placed at -80C overnight and then transferred to liquid nitrogen for storage until use. PBMCs used for macrophage differentiation were immediately plated without freeze down.

To prepare macrophages for co-culture with MSCs, T-175 flasks were coated with 20 mL 2% (w/v) type A gelatin (Fisher Scientific, Cat # G8-500) for 2 hours. The gelatin was then aspirated leaving approximately 1 mL of gelatin solution adsorbed to the flask. Flasks were then allowed to dry at 37°C overnight. Immediately prior to starting the monocyte isolation, 15 mL of CELLstart (Invitrogen, Cat # A1014201) was added to each gelatin coated flask and incubated for 1 hour at 37°C. CELLstart was then removed and 15 mL of PBMC suspension obtained from the PBMC isolation was plated into each of the flasks, and the cell solution was incubated at 37°C for 1 hour to allow monocyte attachment to the flask. Non-adherent cells were then washed out of the flasks with PBS twice. The hM media was prepared with RPMI base supplemented with 10% human AB Serum (VWR, Cat # 45001-062), 10% FBS, 1% L-glutamine, and 1% penicillin-streptomycin. The monocytes were lifted with sterile filtered PBS containing 5 mM EDTA (Quality Biological, Cat # 351-027-721) and resuspended at 1 million cells/mL with hM media supplemented with 25 ng/mL of recombinant human M-CSF (PeproTech, Cat # 300-25) prior to plating.

## Macrophage co-culture

1 million monocytes, isolated as described in the Supplemental Materials and Methods, were resuspended in hM media containing RPMI 1640 (Life Technologies, Cat # 11835030) supplemented with 10% human AB Serum (VWR, Cat # 45001-062), 10% FBS, 1% L-glutamine, 1% penicillin-streptomycin, and 25 ng/mL of recombinant human M-CSF (PeproTech, Cat # 300-25) were added to 24-well plates. Cells were differentiated for 6 days, with a media change after 3 days. After differentiation, macrophages were polarized for 2 days in M1 media containing 50 ng/mL LPS (Sigma, Cat # L6529) and 50 ng/mL IFN-γ (PeproTech, Cat # 300-02) or M2c media containing 10 ng/mL M-CSF and 100 nM of budesonide (Tocris, Cat # 2671), the polarizing media was replaced 2 days prior to the co-culture.

For MSC-macrophage co-cultures, either 100,000 adherent MSCs or five-20,000-cell spheroids were added to M1 or M2c macrophages isolated from PBMC monocytes. Co-cultures were incubated at 37°C for 2 days and then processed for mRNA content and protein secretion of TNF-α and IL-10 as found in the Supplemental Materials and Methods and Supplemental Table 1.

### T cell enrichment

Fresh PBMCs isolated as described above were enriched for CD3+ T cells using a MojoSort Human CD3 T Cell Isolation Kit (Biolegend, Cat # 480021). 60 million PBMCs were washed and resuspended at 1x10^8^ cells/mL in sorting buffer containing 1X PBS with 0.5% (w/v) BSA and 2mM EDTA according to the manufacturer’s protocol. 10 µL Biotin-Antibody cocktail and 10 µL streptavidin nanobeads were added per 10 million cells and incubated for 15 minutes on ice. After 8 minutes in a magnet, the supernatant containing unlabeled cells was collected, the remaining beads were resuspended in sorting buffer, and separated again using a magnet as before to increase T cell yield. The collected supernatant was combined, placed in a new tube, and placed in a magnet for an additional 5 minutes to remove any residual nanobeads. The resultant negatively selected T cells were then counted, stained with CFSE, and plated as described above for PBMCs.

### FBS and heat-inactivated FBS co-culture

The thawed FBS was placed into a 56°C water bath such that the tube was immersed up to the level of the serum. The FBS was heated in the water bath for 30 minutes with agitation every 5 minutes to prevent gelling. After 30 minutes the FBS was removed from the water bath and immediately cooled in an ice bath. After cooling, the heat inactivated FBS (HI-FBS) was used to prepare RPMI culture media (10% HI-FBS, 1% penicillin/streptomycin, and 1% L-glutamine).

As described in the methods, 60,000 adherent MSCs or three 20,000-MSC spheroids were plated with 250,000 CSFE labeled PBMCs activated with Dynabeads. UC4078, UC4373, and UC4477 were each paired with a different PBMC donor. Culture was done in RPMI containing FBS or HI-FBS as described above. Cells were co-cultured for 6 days and then proliferation was analyzed by flow cytometry on an Accuri C6 Plus (BD Biosciences).

### Proliferation analysis

Percent proliferation normalized to the positive control was calculated by the formula: % proliferation sample / % proliferation positive control. For Peak fitting analysis of PBMC proliferation, FlowJo v10 was used. Exported FCS flow cytometry files were fit using the Proliferation Modeling module. An 8 peak model was used with fixed ratio, CV, and background parameters. According to the developer’s instruction, parameters were optimized using the RMS of the model on the PBMC alone with and without Dynabead conditions. The software calculated cells per generation were exported, normalized to total PBMC count, and used to calculate the average generation. Average PBMC generation was calculated by the following equation:


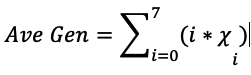


where “i” is the generation multiplied by the fraction of total PBMCs in the “i-th” generation (χ_i_), summed over all PBMC generations (0^th^ to 7^th^ generations).

### PCR for MSC and macrophage mRNA expression

In order to measure changes in inflammatory M1 markers and anti-inflammatory M2 markers, co-cultured macrophages were harvested with 0.4 mL of TRIzol (Thermo Fisher, Cat # 15596018). To collect RNA from adherent MSCs, samples were lysed in 0.4mL of TRIzol and collected. Spheroid MSC samples due to difficultly in disrupting the MSC aggregates, were washed in PBS and then homogenized at 33,000 rpm for 5 seconds using a Tissue Master 125 homogenizer with microprobe attachment (VWR, Cat # TMP125-115). Briefly, to extract mRNA from all samples, each sample lysed in TRIzol was added to 80 μL of chloroform (Sigma Aldrich, Cat # C2432), followed by 12,000g centrifugation for 15 minutes at 4°C. The aqueous layer was collected and 200 μL isopropyl (Sigma Aldrich, Cat # 190764-1L) was added to precipitate the RNA. The mixture was centrifuged at 12,000g for 10 minutes at 4°C. The pellet was then washed with 0.4 mL of 75% (v/v) 200-proof ethanol (Fisher Scientific, Cat # BP2818-500) and centrifuged at 7,500g for 5 minutes. The RNA pellet was air dried for 10 minutes and resuspended with 20 μL DNase- and RNase-free UltraPure water (Life Technologies, Cat # 10977-01). RNA concentrations were determined using NanoDrop (NanoDrop 2000 Spectrophotometer, Thermo Scientific). The mRNA sample was converted into cDNA with a High-Capacity cDNA Reverse Transcription Kit with RNase Inhibitor (Thermo Fisher, Cat # 4374966) as described in the manufacturer protocol. Thermocycler (ProFlex PCR System, AppliedBiosystems) was set to heat the samples at 25°C for 10 minutes, 37°C for 2 hours, 85°C for 5 minutes, and cooled down to 4°C. A 10 μL solution of diluted cDNA (2 ng/mL), POWER SYBR Green Master Mix (Applied Biosystems, Cat # 4367659), primer, and ultrapure water at a ratio of (1:10:1:8) was plated in triplicate into a 348-well PCR plate (Applied Biosystems, Cat # 4309849). The data was analyzed using QuantStudio3 software (AppliedBiosystems) to obtain CT values. All gene expression was normalized to GAPDH expression. A list of primers and catalog numbers is given in Supplemental Table 1.

### Bead ELISA for macrophage/PBMC IL-10/TNF-α/Granzyme B production

To measure cumulative inflammatory and anti-inflammatory factor production, culture media collected from co-cultures was analyzed by bead ELISA and compared to standards of Human TNF (BD Biosciences, Cat # 558273), IL-10 (BD Biosciences, Cat # 558274), and granzyme B (BD Biosciences, Cat # 560304). Capture Beads were mixed with Capture Bead Diluent from the Human Soluble Protein Master Buffer Kit at a ratio of 1:1:48. 50 μL media sample or standard was then added to 50 μL of diluted capture beads and incubated for 1 hour at room temperature. PE Detection Reagents were mixed with Detection Reagent Diluent at a ratio of 1:1:48. After incubation, 50 μL of PE detection mixture was added to each sample and incubated for 2 hours at room temperature, followed by adding 1 mL Wash Buffer. The sample was centrifuged for 5 minutes at 200g. After supernatant removal, each sample was resuspended in 150 μL of the Wash Buffer. The samples for the macrophage assay were then analyzed on an Accuri C6 cytometer (BD Biosciences) and the samples from the PBMC assay were analyzed on the Attune NxT (ThermoFisher).

## Plate ELISA for PBMC co-culture production of IFN-γ/IL-10

Media from PBMC co-cultures were analyzed for IFN-γ and IL-10 content. Briefly, media from PBMC-spheroid co-culture was collected and frozen at -20°C prior to analysis. The day before the analysis, Nunc MaxiSorp ELISA plates (Biolegend, Cat # 423501) were coated with either IFN-γ or IL-10 capture antibody diluted in Coating Buffer (Biolegend, Cat # 421701) and allowed to incubate overnight at 4°C. After the plate was washed 4X with a wash buffer made from PBS containing 0.05% (w/v) Tween-20 (Sigma, Cat # P1379), all wells were blocked using Assay Diluent (Biolegend, Cat # 421203) for 1 hour at room temperature with shaking at 500 rpm on an orbital shaker. Samples for IFN-γ analysis were diluted 20X in Assay Diluent and samples for IL-10 analysis were not diluted. After 4 washes of the plate, 100 μL of samples or standards were added to their respective wells according to the manufacturer’s protocol. Plates were allowed to incubate overnight at 4°C. Plates were treated with their respective capture antibodies and biotin-HRP with appropriate shaking and wash steps as indicated in the manufacturer’s protocol. 100 μL of substrate F (Biolegend, Cat # 437004) was added to each well and allowed to incubate for 30 minutes at room temperature. 50 μL of Stop Solution (Biolegend, Cat # 423001) was added to each well and the absorbance was measured at 450 nm with a background correction wavelength of 570 nm.

### MSC intracellular protein collection

After spheroid formation, adherent and spheroid MSCs from 3 independent preparations of MSCs were cultured for 3 days with or without IFN-γ. MSCs were then washed with PBS and 40 μL of RIPA lysis buffer (Santa Cruz Biotechnology, sc-24948A) was added to each sample. Adherent samples were scraped with a cell scraper (VWR, 89260-222) and spheroid cells were disrupted using a SFX150 Cell Disruptor (Fisher Scientific, Cat # 15-338-528) at a continuous setting of 30% power for 5 seconds. Lysate was spun at 8,000g for 10 minutes at 4°C. After spinning, 5 μL of lysate sample was diluted in PBS to achieve a 1:40 dilution. A 2 mg/mL bovine serum albumin solution (Thermo Scientific, 23235) was diluted 1:1 with PBS to obtain a protein standard curve from 200 to 3.1 μg/mL total protein. 150 μL of diluted lysate or BSA standard were then added to a 96 well plate and 150 μL of working reagent (Thermo Scientific, 23235) was added. Samples were incubated at 37°C for 12 minutes. After incubation, the plate was read at 562 nm on a plate reader (Molecular Devices, 60139412). The BSA standard curve was then used to interpolate the protein concentration in each sample.

### Western Blot

Lysate was denatured by adding 38.5 μL of 4X LDS sample buffer (Thermo Fisher, B0007) and 15.5 μL of 10X Bolt reducing agent (Invitrogen, B0009) per 100 μL of lysate and heated at 95°C for 2 minutes. The gel electrophoresis apparatus (Life Technologies, A25977) was loaded with 4-12% gradient, 10-well bis-tris gels (Bolt, NW04120BOX) and filled with running buffer diluted from 20x stock (Invitrogen, b0002). 10 μg of protein was added for each sample as well as a precision plus protein Kaleidoscope (Bio-Rad, 1610375). The gel was then run at 200V for 20 minutes. Transfer buffer was prepared with 700 mL of milli-Q, 200 mL methanol (Fisher Scientific, A414P-4), and 100 mL 10X Tris-glycine buffer (Amresco, M114-1L). PVDF blotting membrane (GE Healthcare, 10600023) was activated in methanol for two minutes. After running the gel, the gel transferred to the membrane at 10V for 2 hours.

After transfer, the membrane was removed from the cassette and blocked in 5% non-fat dried milk for 1 hour. After blocking, primary antibody was added which consisted of 5% (w/v) BSA (Sigma-Aldrich, A9647-10G), 1:1,000 IDO primary antibody (Cell Signaling, 86630S), 1:10,000 β-actin primary antibody (Thermofisher Scientific, AM4302), and 1:250 COX-2 primary antibody (BD Biosciences, 610203). Primary antibody staining was performed overnight at 4°C with gentle rocking. After primary antibody staining, the appropriate secondary antibody was then added and incubated at room temperature. 1:10,000 Goat anti-rabbit antibody (BD Pharmingen, 554021) was used for IDO while 1:5,000 goat anti-mouse antibody (BioLegend, 405306) was utilized for COX-2 and β-actin. HRP substrate, a WesternBright Quantum mix (Advansta, K-12042-D10), was added to the membrane and scanned on a C-DiGit Blot Scanner (LI-COR Biosciences, 3600-00).

### Kynurenine measurement

In order to measure IDO enzymatic activity, we directly measured conversion of tryptophan to kynurenine. Cells were cultured in 250 μM Tryptophan supplemented MEM-α with or without 100 ng/mL rhIFN-γ for 3 days after which media was collected and used in the activity assay. A 1 mM kynurenine stock was diluted 1:1 with media to obtain a standard curve ranging from 200 μM to 3.1 μM. 200 μL of kynurenine standards or media samples were then added to a 96 well plate. 100 μL of 30% (w/v) trichloroacetic acid (Sigma-Aldrich, T9159-100G) was added to each active well to precipitate proteins. The plate was then heated at 52°C for 30 minutes to convert N-formylkynurenine to kynurenine. After heating, the plate was spun at 1,200g for 15 minutes. 75 μL of supernatant was then collected and mixed 1:1 with 0.8% (w/v) p-dimethylaminobenzaldehyde (Sigma-Aldrich, 156447-25G). Absorbance was measured on a plate reader at 492 nm. Absorbance values of the known kynurenine standards were used to interpolate media kynurenine concentrations.

### PGE2 ELISA

The concentration of Prostaglandin E2 (PGE2) in cell culture media was determined by Prostaglandin E2 Human ELISA Kit (Life Technologies, Cat # KHL1701) as described in the manufacture’s protocol. Briefly, the standard was prepared by serial 1:1 dilutions from 2,500 to 39.1 pg/mL. Calibrator diluent alone was added as a non-specific binding control. 150 μL of standards or samples were added into pre-coated 96 well plate. 50 μL of primary antibody solution was added to all wells except the well for non-specific binding, followed by a 1-hour incubation at room temperature on orbital shaker with 500 rpm. After incubation, reagents were aspirated, and each well was washed with 300 μL of Wash Buffer 4 times. 200 μL substrate solution was then added to all wells, followed by incubation at room temperature for 30 minutes in dark. 100 μL of stop solution was added to every well after incubation and the plate was read at 570 nm and 450 nm to perform wavelength correction.

### CD73 expression and activity

To evaluate CD73 expression at cell surfaces, adherent cells were lifted using Accutase. Spheroid MSCs were collected, washed in PBS, and incubated with Accutase at 37°C. Spheroids were incubated for 4 minutes, followed by vigorous pipetting to disrupt the spheroids. The suspension was incubated at 37°C for another 4 minutes to ensure complete dissociation of spheroids. 200,000 cells from both adherent and spheroid MSC suspensions were spun down and resuspended in 100 μL of Cell Staining Buffer. For each sample, 5 μL of PE-Cy7 anti-CD73 (BD Biosciences, Cat # 561258) or 5 μL of PE-Cy7 Mouse IgG1K Isotype Control (BD Biosciences, Cat # 557872) was added. The samples were incubated on ice for 30 minutes, followed by flow cytometry analysis. Reported CD73 MFI values were baseline corrected using the MFI of the isotype control.

To evaluate CD73 expression in both adherent MSCs and spheroid MSCs, 20,000 cells per well were plated in either standard or spheroid (Corning, Cat # 4515) 96-well plates, respectively, with 100 μL of MEM-α complete. After culturing for 3 days, media was swapped to 75 μL of base MEM-α supplemented with 25 μL 1.2 M AMP (Fisher Scientific, Cat # 50-490-075), followed by incubation at 37°C for 48 hours. The culture media was then collected for free phosphate measurement. Malachite Green Assay (Sigma Aldrich, Cat # MAK307-1KT) was performed as described in manufacturer protocol to quantify free phosphate. Briefly, reagent A and B were mix with ratio of 100:1 to form working reagent. 80 μL of 400x diluted sample and 20 μL of working reagent were added to a 96-well plate. The plate was incubated at 37°C for 30 minutes, followed by measurement of absorbance readings at 620 nm and 800 nm on a plate reader. 800 nm readings were used for background subtraction.
