# Supplementary material for: Aggregation of Human Mesenchymal Stromal Cells Eliminates Their Ability to Suppress Human T Cells
Source: Front Immunol. 2020 Feb 25;11:143. doi: 10.3389/fimmu.2020.00143 (PMC7052295; doi:10.3389/fimmu.2020.00143)
Supplement: Supplementary file 2 [file Data_Sheet_2.pdf]

Supplementary Material

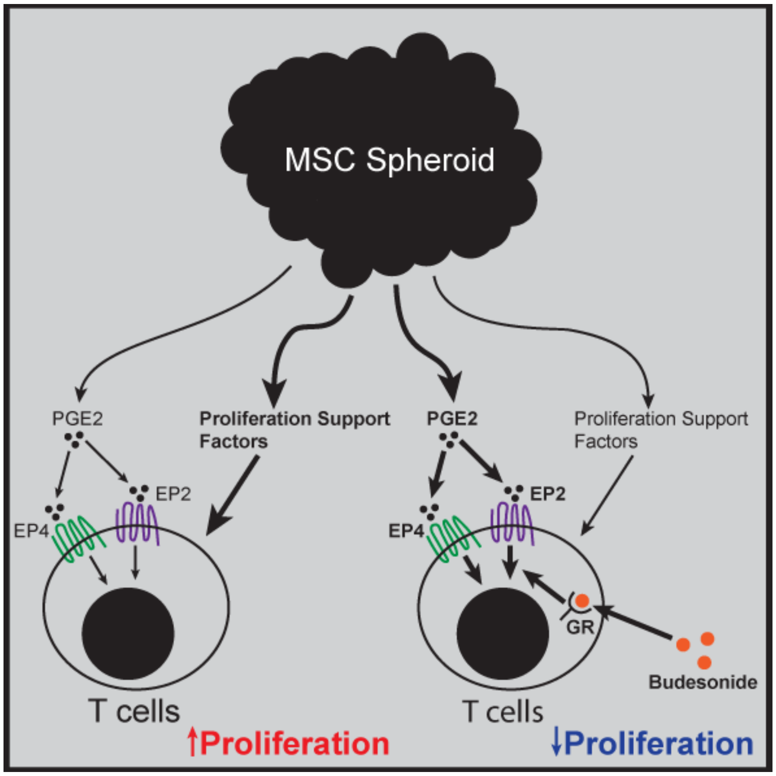

Graphical Abstract

Aggregation of human mesenchymal stromal cells (MSCs) into spheroids perturbs the balance of immunomodulatory factors produced by MSCs ultimately leading to a loss in the suppression of activated human T cells. While spheroid MSC produced PGE2 is not alone suppressive, when combined with budesonide, a glucocorticoid steroid, it works in synergy to inhibit the proliferation of activated T cells.

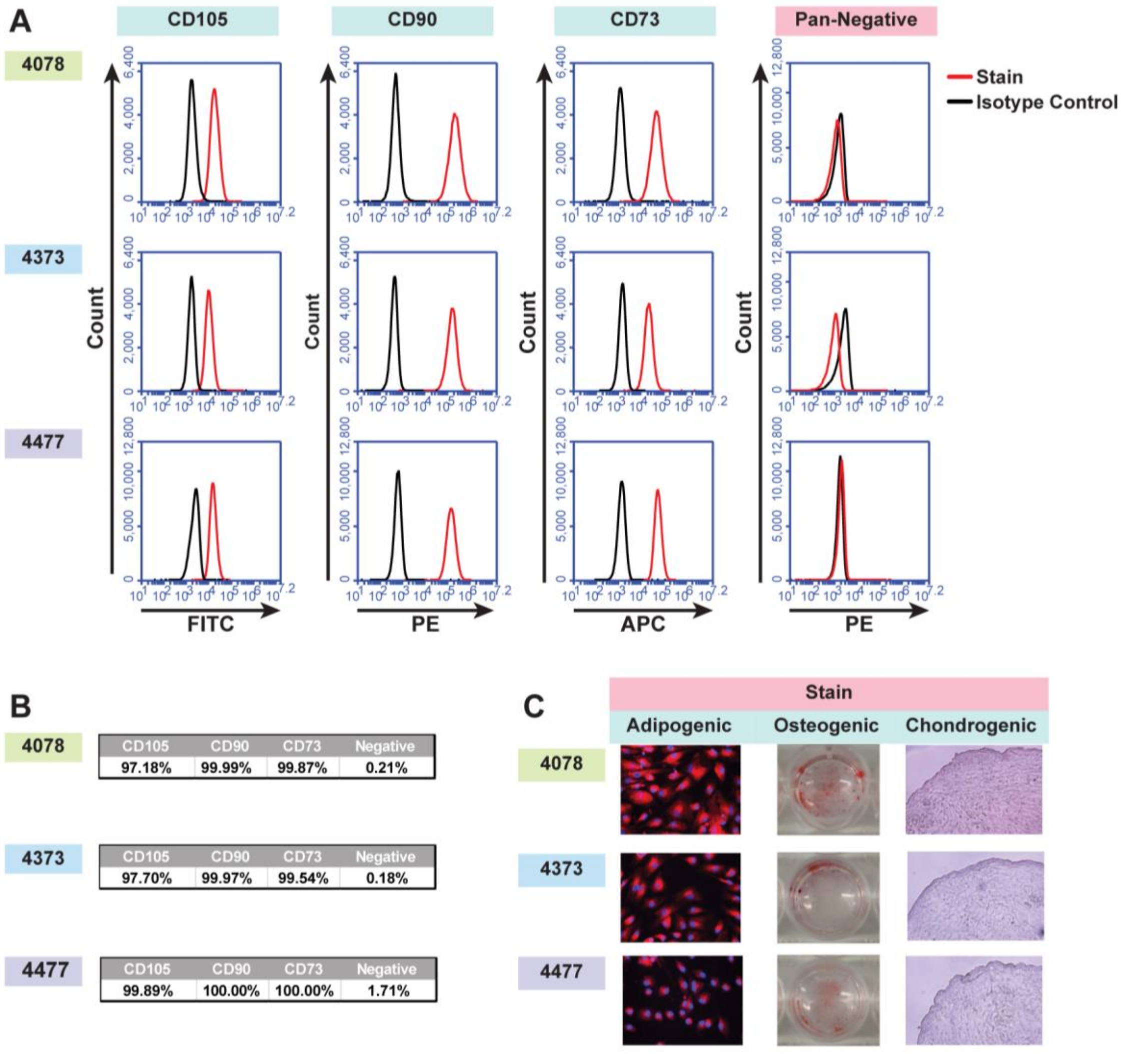

Supplemental Figure 1. Isolated umbilical cord cells meet the minimal MSC criteria. (A) Flow cytometry analysis of MSC markers CD105, CD90, CD73, and negative cocktail (CD34, CD11b, CD19, CD45, and HLA-DR) for isolated umbilical cord MSCs. (B) Quantification of MSC marker expression in (A). (C) Differentiated umbilical cord MSCs stained with AdipoRed, Alizarin Red, and Safranin-O for confirmation of adipogenic, osteogenic, and chondrogenic differentiation potential.

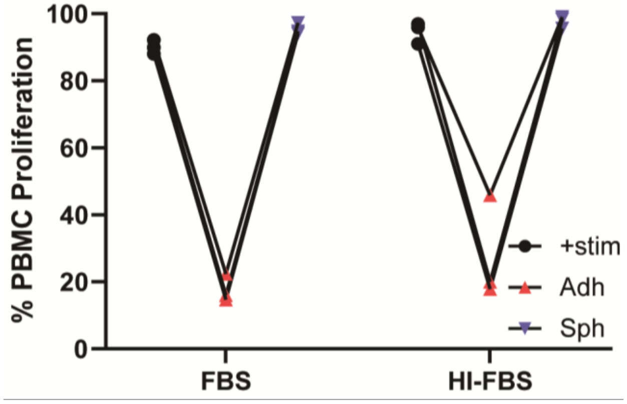

Supplemental Figure 2. Heat-inactivation of FBS does not restore spheroid MSC suppression of PBMCs. 3 unique pairings between 3 independent PBMC donors and 3 umbilical cord MSCs (UC4078, UC4373, and UC4477), as either 60,000 adherent MSCs or spheroids, were cultured in RPMI containing 10% FBS or 10% heat inactivated FBS. PBMC proliferation was analyzed after 6 days in culture. 2-way ANOVA revealed no significant effect of Heat Inactivation alone or in interaction with the presence or absence of MSCs.

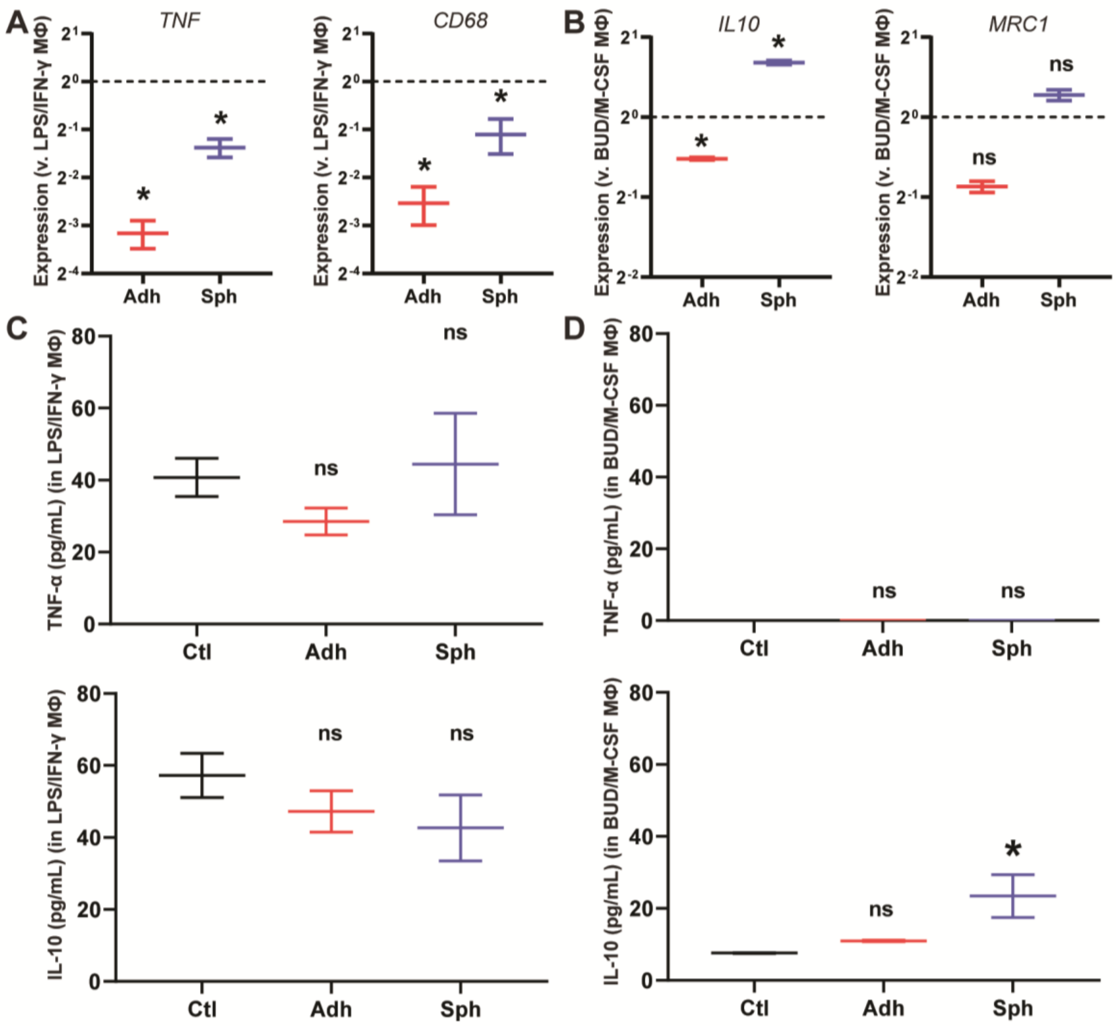

Supplemental Figure 3. Spheroid MSCs alter macrophage phenotype. (A) Gene expression of primary human macrophages differentiated with LPS/IFN-γ (M1 like) normalized to the no cell control (indicated by the dashed line). (B) Gene expression of primary human macrophages differentiated with M-CSF/BUD (M2c like) normalized to the no cell control (indicated by the dashed line). (C) TNF-α and IL-10 bead ELISA data from the co-culture conditioned media in (A). (D) IL-10 bead ELISA data from the co-culture conditioned media in (B). Data was analyzed with One-Way ANOVA with Sidak correction for multiple comparisons.

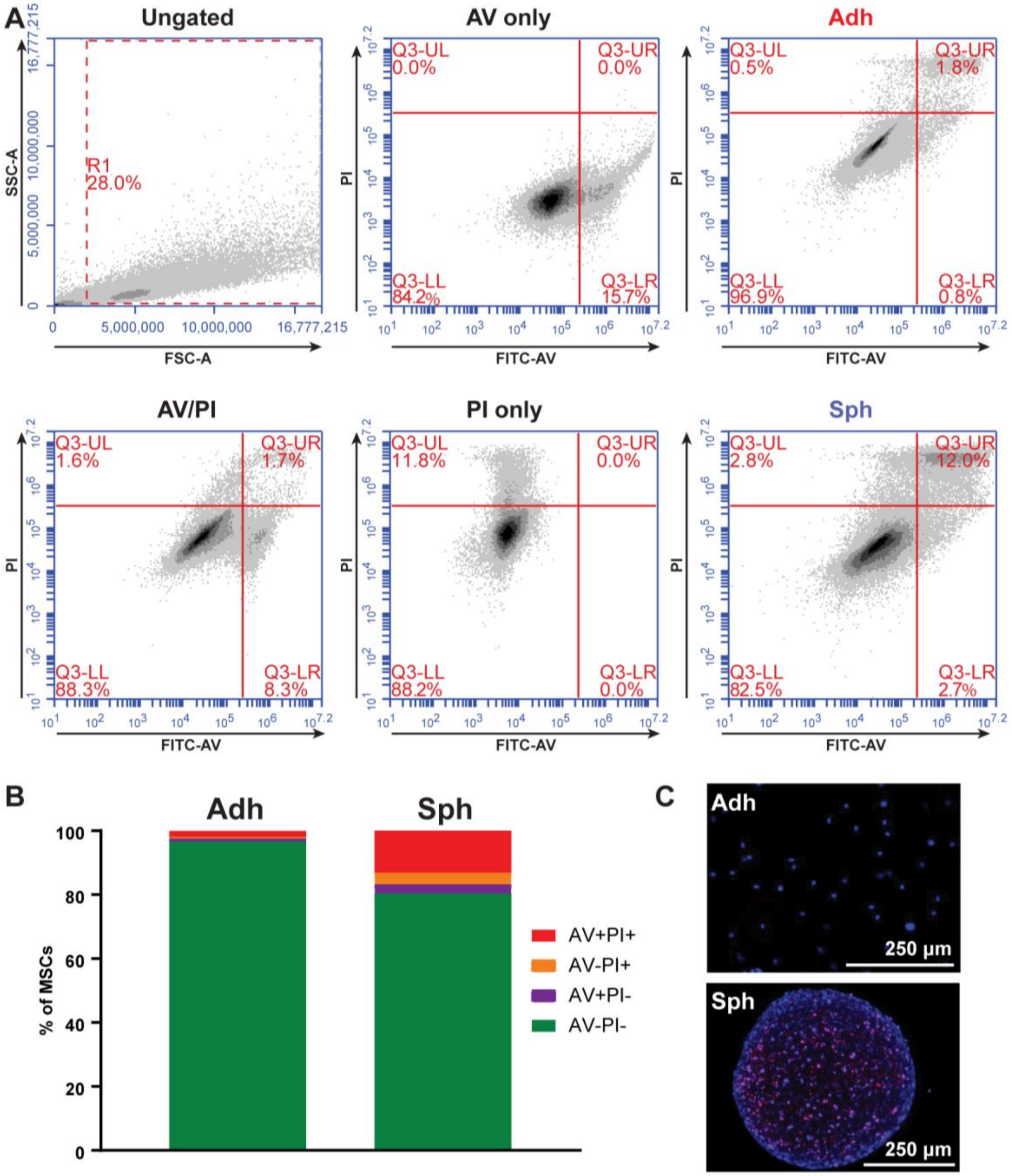

Supplemental Figure 4. Spheroid MSCs show decreased viability compared to adherent MSCs. (A) Representative flow cytometry plots for annexin/PI stained adherent and spheroid MSCs. AV only, PI only, and AV/PI controls were run using staurosporine treated adherent cells. (B) Quantification of annexin (AV) and PI staining from flow cytometry analysis in (A). (C) Representative images of adherent or spheroid MSCs stained with PI and Hoechst.

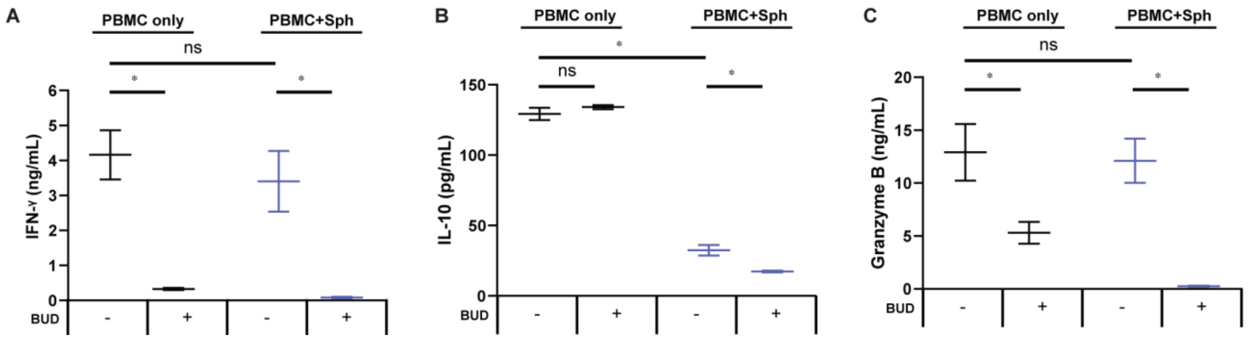

Supplemental Figure 5. Spheroid MSCs with budesonide affect PBMC production of cytokines. Media collected from Figure 4C co-culture was assayed for IFN-γ (A), IL-10 (B), and granzyme B (C). Statistics were performed using a Two-Way ANOVA with Tukey correction for multiple comparisons. *p<0.05

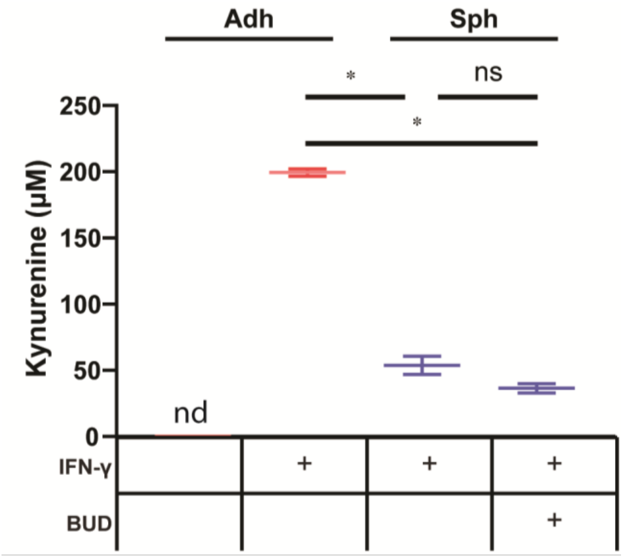

Supplemental Figure 6. Budesonide treatment of spheroid MSCs does not restore kynurenine production. 20,000-cell spheroid MSCs were treated with 250 μM tryptophan and 100 ng/mL IFN-γ with or without 10 μM budesonide for 72 hours. Media was collected and analyzed for kynurenine. Adherent MSCs with or without 100 ng/mL IFN-γ served as positive and negative controls. Statistics were performed using a One-Way ANOVA with Tukey correction for multiple comparisons. *p<0.05

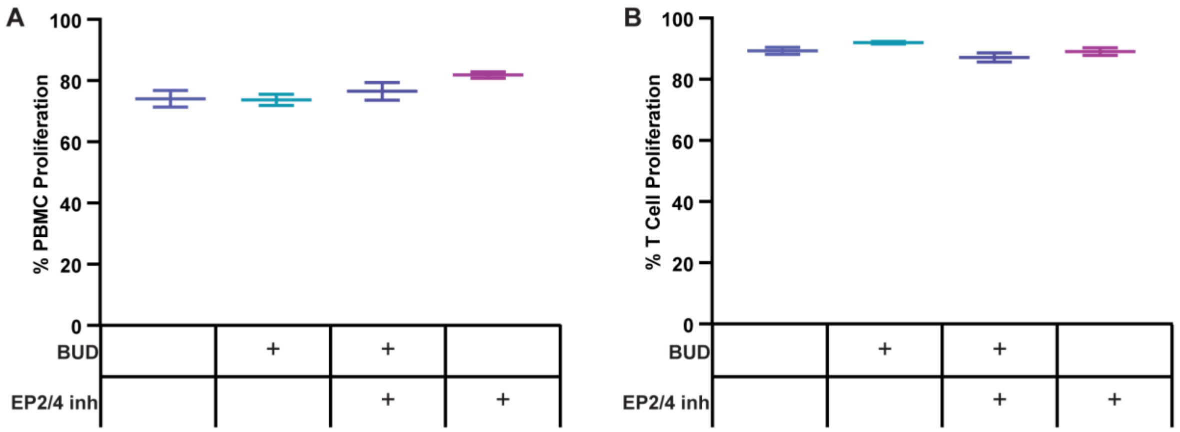

Supplemental Figure 7. Budesonide and EP inhibitors do not affect PBMC proliferation alone. (A) Flow cytometry proliferation analysis of PBMC alone with 10 μM budesonide and/or 10 μM TG4-155/L-161,982 targeting EP2/4 (N=3 PBMC donors). (B) Flow cytometry analysis of isolated T cell proliferation with drugs as in (A) with N=3 PBMC donors. Statistical analysis was performed using a One-Way ANOVA with Sidak correction for comparison to the no drug control. *$p<0.05$
